# Wnt signaling in gastric cancer: current progress and future prospects

**DOI:** 10.3389/fonc.2024.1410513

**Published:** 2024-06-07

**Authors:** Ruyue Han, Jing Yang, Yingying Zhu, Runliang Gan

**Affiliations:** ^1^Cancer Research Institute, Key Laboratory of Cancer Cellular and Molecular Pathology in Hunan, Hengyang Medical School, University of South China, Hengyang, Hunan, China; ^2^Department of Gastroenterology, The First Affiliated Hospital of University of South China, Hengyang Medical School, University of South China, Hengyang, Hunan, China

**Keywords:** gastric cancer, Wnt, EMT, tumor microenvironment, molecular agents

## Abstract

Levels of the Wnt pathway components are abnormally altered in gastric cancer cells, leading to malignant cell proliferation, invasion and metastasis, poor prognosis and chemoresistance. Therefore, it is important to understand the mechanism of Wnt signaling pathway in gastric cancer. We systematically reviewed the molecular mechanisms of the Wnt pathway in gastric cancer development; and summarize the progression and the challenges of research on molecular agents of the Wnt pathway.

## 1 Introduction

Gastric cancer(GC) has the fifth highest incidence and fourth highest mortality rate in the worldwide (1). Activation of Wnt1 was first identified more than 40 years ago as causing breast hyperplasia and breast tumors, thus linking the Wnt pathway to cancer (2). Recent studies have identified frequent dysregulation of Wnt pathway activity in GC, and the phenomenon is associated with proliferation and metastasis, tumor microenvironment regulation, stemness maintenance, treatment resistance, and prognosis of gastric cancer (3–6). Inhibitors acting on different components of the Wnt pathway have been continuously developed, and some of them have shown good anticancer potential (7).

## 2 The Wnt signaling pathway

**Figure 1:**
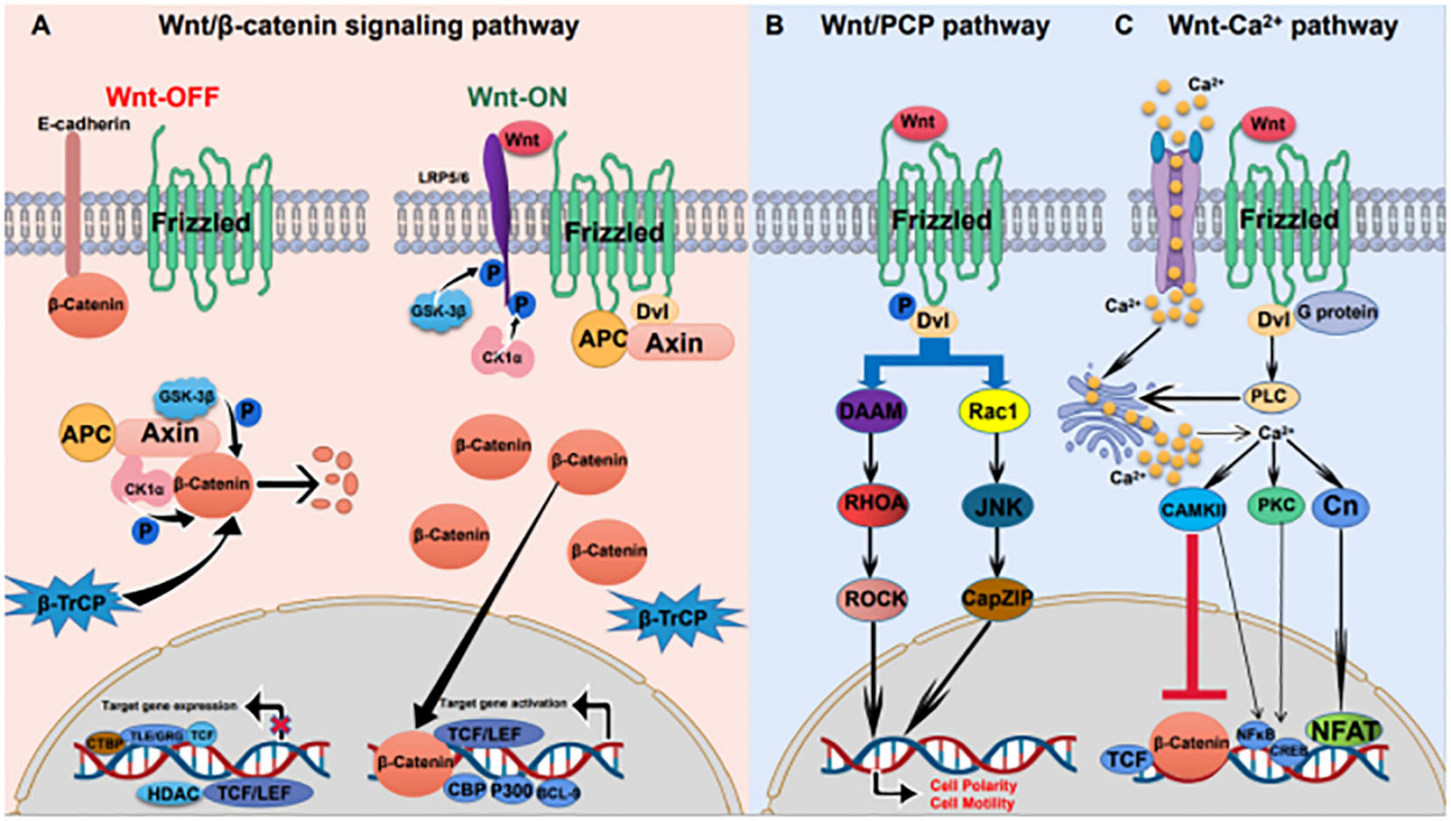
Wnt signaling pathway in gastric cancer **(A)** Wnt/β-catenin signaling pathway: in the absence of a Wnt ligand, β-catenin is degraded by the destruction complex; in the presence of a Wnt ligand, Wnt ligand binds to the receptor, and β-catenin separates from the destruction complex and is transported to the nucleus, resulting in the expression of target genes. **(B)** Wnt/PCP pathway: Wnt ligand binds to the Fzd receptor and phosphorylates Dvl. Phosphorylated Dvl activates RHOA and Rac1, causing downstream cascade reactions. **(C)** Wnt/Ca^2+^ pathway: Increased intracytoplasmic Ca^2+^ activates the protein phosphatase Cn, PKC, and CAMKII. CaMKII and PKC activate NF-κB and CREB. Cn activates nuclear factor of T cells (NFAT).

Wnt signaling pathways include the classical Wnt/β-catenin pathway, the non-classical Wnt/PCP pathway and the Wnt/Ca^2+^ pathway (**Figure 1**). Wnt ligands bind to the transmembrane receptor Frizzled (Fzd) to activate the Wnt pathway (8). The classical Wnt pathway is mainly involved in cell proliferation, while nonclassical Wnt pathways participate in cell polarity and migration (9).

## 3 Altered Wnt signaling in GC Cells

Wnt ligand expression levels, β-catenin cytoplasmic and nuclear content, and intracellular cytoplasmic Ca^2+^ concentration were all altered. More than half of all gastric cancer patients have significant dysregulation of the Wnt/β-catenin pathway (5). The number of patients with gastric carcinogenesis with only nonclassical Wnt pathway activation is much higher than that of patients with only classical Wnt pathway activation (10).

### 3.1 Wnt ligand

Wnt ligands constitute of 19 members with functions that are largely dependent on posttranscriptional modifications, including glycosylation, palmitoylation and acylation. Among these modifications, acylation is necessary for extracellular transport of Wnt ligands and receptor/coreceptor recognition and binding (11), and Wnt ligands are essential for activating the Wnt pathway signaling cascade, making these ligands attractive therapeutic targets in gastric cancer. The vast majority of Wnt ligands show altered expression levels in gastric cancer cells, which affects the growth phenotype these cells and the efficacy of chemotherapy (11).

#### 3.1.1 WNT1

In 2022, Zheng et al. found that high expression of *WNT1* ligands in GC not only lead to malignant proliferation, invasion, and migration of gastric cancer cells but also accelerates the self-renewal and proliferation of GC stem cells (GCSCs) (12). LINC00665 activates *WNT1* by binding to the *WNT1* promoter, leading to M2 polarization of tumor-associated macrophages and promoting GC progression. It has been suggested that *WNT1* is involved in gastric cancer progression and the induction of immune tolerance (13).

#### 3.1.2 WNT2

Immunohistochemistry assays have confirmed that WNT2 was positively expressed only in gastric cancer tissues (14). The high expression of *WNT2* was related to β- Catenin entering the nucleus. Collagen type X alpha 1 (COL10A1) activates the *WNT2*-mediated Wnt pathway and promotes the development of gastric cancer (15), and the vast majority of patients with high expression of *WNT2* have cancer in TNM Stage III or IV with lymph node metastasis, it may be a marker for patients with advanced GC (14). *WNT2B* is a paralog of *WNT2 (* 16). In 2022, Zhang et al. found that *WNT2B* is an oncogene in GC and upregulated in both GC tissues and GC cell lines (17). The mRNA of *WNT2B* carries a binding site for microRNA- 376c-3p and that overexpression of microRNA-376c-3p reduces the expression of *WNT2B*, inhibiting the growth and promoting the apoptosis of gastric cancer cells (17).

#### 3.1.3 WNT3

An immunohistochemical positive assay indicated that the expression of WNT3 reached 87.50% of the cells in the poorly differentiated carcinoma group, which was higher than that in the precancerous lesion group (61.11%) and the chronic superficial gastritis group (20%) (18). Interference with *WNT3* expression suppresses the malignant phenotype and promotes apoptosis in gastric cancer cells (19). *WNT3A* is a homolog of *WNT3*, with an amino acid homology as high as 84.2% (20). As a procarcinogenic gene in gastric cancer tissues, WNT3A expression is nearly twice that in adjacent noncancerous tissues (21), not only promoting malignant proliferation of gastric cancer cells and inhibiting apoptosis but is also patient chemoresistance and gastric cancer neuroinvasion (21, 22). It promotes gastric cancer cell proliferation by activating the downstream target high mobility Group A type 1 protein (HMGA1) (23). High expression of *WNT3A* is closely associated with neural infiltration in gastric cancer patients and may be caused by activation of matrix metalloproteinases (MMPs) downstream of the Wnt/β-catenin pathway (21). Therefore, *WNT3A* may be a potential prognostic indicator, although a large amount of clinical data are needed to validate its usefulness as a biomarker.

#### 3.1.4 WNT4

Using a database for gene expression analysis of gastric cancer, in 2013, Volkomorov et al. (24) found that one-half of gastric cancer patients presented with a greater than twofold reduction in *WNT4* expression compared to the level in normal gastric epithelial tissues. The main active metabolite, indole-3-methanol, 3,3′-diindolylmethane (DIM), prevents and treats cancers by inhibiting cell proliferation and inducing apoptosis; however, low-dose DIM induced *WNT4* secretion and promoted gastric cancer progression (25).

#### 3.1.5 WNT5A

The immunohistochemical assay showed that the *WNT5A* expression was evident in approximately 77.6% of gastric cancer tissues, and *WNT5A*-positive gastric cancer patients showed a higher infiltration of late tumor cells and more lymph node metastases than patients who did not express *WNT5A*. These differences might be related to the epithelial-mesenchymal transformation(EMT) of gastric cancer cells induced by *WNT5A*. Notably, the five-year survival rate was lower for *WNT5A*-positive patients in *WNT5A*-negative patients (26). *WNT5A* activates the nonclassical Wnt/Ca^2+^ pathway by binding to the *FZD2* and promotes the invasion and migration of gastric cancer cells (27). In 2019, Xu et al. (28) reported that inhibition of zinc-finger transcription factor 1 (ZEB1) reduced the level of *WNT5A* in GC, induced apoptosis and inhibited the proliferation and migration of GC cells. Current research on *WNT5B* is not very clear, although it can predict the sensitivity of the epigenetic regulator BET inhibitor iBET-151 to inhibit the growth of gastric cancer, especially when combined with paclitaxel (29). Therefore, it may be possible to predict the effect of chemotherapy in gastric cancer by *WNT5B*.

#### 3.1.6 WNT7、WNT8

The expression of *WNT7A* in gastric cancer is controversial. One study showed that its expression was decreased in gastric cancer tissues and gastric cancer cell lines, and the immunohistochemistry positivity rate of gastric cancer tissues was only 51.3%, whereas the positivity rate of normal gastric mucosal tissues was as high as 86.7% (30), and its low expression often suggests that the prognosis of gastric cancer patients is poor; another study showed that *WNT7A* elevated expression, which promotes migration and invasion of gastric cancer cells. miRNA-127 can target the 3′-UTR region of *WNT7A* mRNA, reduce its expression, and inhibit the invasion and migration of gastric cancer cells (31). *WNT7B* shares 77.1% amino acid homology with *WNT7A* (32). Moreover, Serum levels of *WNT7B* are higher in gastric cancer patients with lymph node metastasis than in gastric cancer patients without lymph node metastasis (33), and the level of serum *WNT7B* was positively correlated with the progression and metastasis of GC. It has been suggested that the effect of treatment can be observed and the prognosis of patients can be evaluated by measuring serum *WNT7B* levels.

The total amino acid homology of the *WNT8A* and *WNT8B* genes was 60.0%, and human *WNT8A* mRNA was expressed in NT2 cells with neural differentiation potential, whereas human *WNT8B* mRNA was expressed in diffuse gastric cancer (34).*WNT8B* was significantly up-regulated in gastric cancer cell lines.*WNT8B* may play a critical role in gastric cancer by activating the β-catenin-TCF signaling pathway (35).

#### 3.1.7 WNT10

The expression of *WNT10B* was up-regulated. Down-regulation of *Wnt10B* expression using shRNA inhibited EMT in cancer cells. stem cell marker Oct4 was positively correlated with the expression level of *WNT10B* in gastric cancer tissues, and *Wnt10B* may also be involved in the generation and maintenance of gastric cancer stem cells (36).

#### 3.1.8 WNT11, WNT16

*WNT11*, an important ligand in the nonclassical Wnt pathway, can promote tumorigenesis (37). In 2016, Mori et al. (38) found that hypoxia and hypoxia-inducible Factor 1α induced *WNT11* expression, increased the activity levels of matrix metalloproteinase (MMP)-2 and 9, and promoted the proliferation, migration and invasion of gastric cancer cells.

As a Wnt family members, *WNT16* can lead to gastric cancer chemoresistance through paracrine secretion, and the polyphenolic flavonoid compound quercetin (QC) can decrease the expression of *WNT16*. In 2018, Fang et al. (39) developed a hyaluronic acid (HA)-modified silica nanoparticle (HA-SiLN/QD) that reversed chemoresistance and remodeled tumor microenvironments by recognizing the overexpression of CD44 in gastric cancer cells and specifically targeting the delivery of quercetin and the gastric cancer chemotherapeutic agent adriamycin (DOX).

### 3.2 Wnt receptors and coreceptors

Aberrant expression of Wnt family transmembrane receptors and their coreceptors leads to overactivation of the Wnt pathway and affects gastric cancer development. In this section, we outline the roles of the Fzd receptor, coreceptors LRP and ROR1/2 and related molecules affecting the expression of these receptors and coreceptors in gastric cancer.

#### 3.2.1 FZD

*FZD* proteins are seven-transmembrane coiled-coil receptors of which 10 (*FZD1-FZD10*) have been identified in humans. The cysteine-rich structural domain (CRD) of the extracellular N segment binds to Wnt ligands, and the intracellular C-terminus binds to Dvl to activate the downstream cascade reaction. *FZD* exerts both tumor-promoting and tumor-suppressing effects on gastric cancer cells. With the exceptions of *FZD3* and *FZD6*, which are expressed at low levels in gastric cancer, all *FZD* isoforms are highly expressed in gastric cancer cells (40–46). *FZD1* and *FZD8* as oncogenes promote the proliferation, invasion, and migration of gastric cancer cells (40), and high expression of *FZD8* suggests that a patients has a poor prognosis (41). *FZD2* and *FZD7* can be used as markers to assess the treatment and prognosis of gastric cancer patients and guide individualized treatment of patients with GC. The *FZD2* level can be used to predict median overall survival (OS), the degree of immune cell infiltration, and the immune response of gastric cancer patients and to assess the effect of immunotherapy, with *FZD7* expression suggesting a higher gastric cancer incidence and lymph node metastasis (42, 43). Moreover, knockdown of *FZD7* decreased the expression of the stem cell genes Nanog and Oct-4, the multidrug resistance transporter protein gene ABCG2, and the tumor stem cell-associated surface antigens CD133, CD44, and CD24, indicating that *FZD7* possibly involved in the regulation of gastric cancer stem cell function and drug resistance (46). In contrast to other isoforms, *FZD5* and *FZD6* play anti-oncogenic roles in gastric cancer. *FZD5* maintains the epithelioid phenotype of gastric cancer cells, inhibits EMT of gastric cancer, and reduce its metastatic potential (44). *FZD6* inhibits the proliferation and migration of gastric cancer cells (45).

Wnt pathway often engages in crosstalk with other pathways to affect the development of GC: it engages in crosstalk with the TGF-β pathway to inhibit the— apoptosis of gastric cancer cells. The transcription factor Smad family member 4 (SMAD4), a core factor in the TGF-β signaling pathway, increases the transcriptional activity of *FZD4* and activates the Wnt pathway (47); *FZD10* promotes the proliferation of gastric cancer cells. Moreover, the expression of *FZD10* is increased in plasma exosomes, *FZD10* can be used as a plasma biomarker (48).

#### 3.2.2 LRP5/6, LGR5

*LRP5/6* function as important coreceptors in the Wnt pathway. *LRP5* is highly expressed; its high expression suggests late clinical stage and poor prognosis of patients with gastric cancer (49). In 2022, Zheng et al. (50) found that *LRP6* interacted with capillary morphology genesis gene 2 (CMG2) to maintain the stemness of gastric cancer stem cells and accelerate gastric cancer progression.

The leucine-rich repeat sequence G protein-coupled receptor 5 (*LGR5*), a seven times transmembrane receptor. It’s a marker for gastric cancer stem cells, and its expression is higher in intestinal-type gastric cancer than in diffuse-type GC. In 2022, Nakazawa et al. (51) demonstrated that LGR5 expression in intestinal-type gastric cancer indicated poor overall survival (OS) (52).

### 3.3 β-Catenin

β-Catenin is a key component of the Wnt pathway, and immunohistochemistry assays that showed high β-Catenin levels in the group with poorly differentiated gastric adenocarcinomas was as high as 90.63%, which was much higher than that in the chronic superficial gastritis group and the precancerous group (18). In 2020, Ye et al. (53) found that the expression of myosin heavy chain 9 (MYH9) was increased in metastatic gastric cancers and was correlated with a poor prognosis; MYH9 provides gastric cancer cells with the ability to resist apoptosis and promote metastasis by inducing the transcription of CTNNB1.The expression of the destruction complex member APC is downregulated in GC, leading to lymph node metastasis and distant metastasis of GC (54). The role of RAS-related C3 botulinum toxin substrate 1 (Rac1) is mediation of β-catenin nuclear localization, and its activation promotes the proliferation, invasion, and drug resistance of gastric cancer cells (55).

Ca^2+^ is a key component of the nonclassical Wnt/Ca^2+^ pathway, and an elevated intracellular Ca^2+^ concentration can activate or inhibit β-catenin action through different pathways. Plasma membrane Na+/Ca^2+^ exchanger 1 (NCX1) and transient receptor potential canonical (TRPC1) play a key role in maintaining cytosolic free Ca^2+^ homeostasis. Elevation of Ca^2+^ levels induced by the coupling of these two proteins increases β-catenin phosphorylation (Ser675), and phosphorylated β-catenin enters the nucleus to activate downstream effector molecules and promote H. pylori-associated GC cell proliferation, migration and invasion (56). However, in 2016, Wan et al. (57) used nucleotide UTP to activate the P2Y6 receptor, and the elevation of cytoplasmic Ca^2+^ concentration in gastric cancer cells induced by store-operated calcium entry (SOCE) inhibited β-catenin, thus inhibiting the proliferation of gastric cancer cells. Moreover, this inhibitory effect did not alter the proliferation of normal gastric cells, making it a potential strategy for preventing or treating GC (58).

Wnt ligand, FZD receptor, and β-catenin are core components of the Wnt pathway, and their expression is altered to varying degrees in gastric cancer and affects the progression and prognosis of gastric cancer patients. The development of specific drugs targeting all three of them may achieve unexpected efficacy in the treatment of gastric cancer patients.

## 4 Wnt signaling in the microenvironment of gastric cancer

For the last few years, the emergence of immune checkpoint inhibitors (ICIs) has brought new hope to gastric cancer patients. Understanding the mechanism of tumor microenvironment (TME)-induced immune tolerance in gastric cancer will help to overcome the therapeutic inefficacy for patients with advanced gastric cancer. Gastric cancer cells establish a complex TME, in which immune cells, the extracellular matrix (ECM), and cell-secreted cytokines are important components (59). The Wnt/β-catenin pathway can inhibit T-cell infiltration and reduce the sensitivity of gastric cancer cells to anti-PD-1 antibodies (60). In 2020, CCL28, a mucosal-associated epithelial chemokine, is the key factor in the activation of β-catenin/TCF directly targeted genes, which can increase the infiltration of regulatory T (Treg) cells and lead to immunosuppression in the gastric cancer microenvironment (61). During the progression of gastric cancer, increased ECM deposition disrupts the interaction between E-cadherin and β-catenin and promotes gastric cancer cell proliferation, invasion and metastasis (62). WNT ligands (e.g., WNT2, WNT5A) from tumor cells and cancer-associated fibroblasts (CAFs) in the TME activate the Wnt pathway through the proliferation of CSCs and resurrection of dormant CSCs, causing treatment resistance and cancer recurrence (63). In 2020, Maeda et al. (64) found that H3K27me3 deletion in CAFs promoted the secretion of WNT5A, which may have enhanced the invasiveness of GCSCs. In 2016, Tumor-associated macrophages (TAMs) in TMEs secrete the proinflammatory factor TNF-α to activate the Wnt pathway and thus promote gastric cancer development (57). In 2016, Bone marrow-derived mesenchymal stem cells (BM-MSCs), important components of the TME, have been shown to promote the proliferation of gastric cancer cells through Wnt5a-Ror2 pathway (65). The evidence presented suggests that the Wnt pathway is related to immunosuppression in GC, and combining Wnt inhibitors and immunotherapy may lead to increased therapeutic effects (3).

## 5 Wnt signaling regulates stemness and drug resistance in gastric cancer cells

GCSCs, key players in gastric cancer with self-renewal and tumorigenic potential, are involved in both chemoresistance and cancer metastasis and gastric cancer recurrence. WNT1 is involved in the maintenance and proliferation of GCSCs through the classical Wnt pathway (66), and WNT5A is involved in the initiation of the EMT and gastric cancer stem cell proliferation through the nonclassical Wnt pathway (67, 68). Overexpression of human epidermal growth factor receptor-2 (HER2) in GC activates Wnt/β-catenin pathway in GCSCs, leading to enhanced invasiveness and treatment resistance of gastric cancer cells (69). In 2021, Wen et al.found that The retinoic acid-associated orphan receptor β (RORβ) reduces the activity of the Wnt/β-catenin pathway in GCSCs to inhibit their tumor-forming ability (70). The homology cassette transcription factor NANOG is essential for embryonic stem cell renewal, and the related pseudogene NANOGP8 is a major contributor to NANOG-induced effects on gastric cancer. NANOGP8 has been associated with almost all malignant phenotypes of GC: it has been associated with the upregulation of GCSC markers and EMT-related genes, accumulation of β-catenin in the nucleus, enhancement of Wnt signaling, and increased gastric cancer cell drug resistance; NANOGP8 is a potential therapeutic target for gastric cancer (71).

Aberrant activation of glutathione peroxidase 4 (GPX4), a key antioxidant enzyme, confers resistance to radiation and chemotherapy-induced iron death to cancer cells (72). The β-catenin/TCF4 transcriptional complex in the Wnt pathway directly binds to and induces the expression of GPX4, which attenuates the production of cytosolic lipid ROS, leading to iron death resistance (29). H. pylori infection upregulates GPX4 expression and activity through TCF4, leading to a high antioxidant state of cancer cells and inhibition of iron-induced death; thus, GPX4 may be a potential target to enhance chemosensitivity in patients with advanced GC (29).

Both multidrug resistance (MDR)-related proteins and Wnt/β-catenin pathway markers were upregulated in gastric cancer drug-resistant cells, overexpression of the novel tumor suppressor basic leucine zipper ATF-like transcription factor 2 (BATF2) inhibited the Wnt/β-catenin pathway to improve drug sensitivity and attenuate gastric cancer drug resistance (73). TRIM14 is a member of the triple structural domain protein (TRIM) family and plays an E3 ubiquitinating enzyme role. In 2023, Chen et al. (74) demonstrated *in vitro* and *in vivo* that circ_0091741 increased the expression of TRIM14 by blocking the binding of miR-330–3p to TRIM14, which activated Wnt/β-catenin by stabilizing the Dvl2 pathway to promote chemoresistance in gastric cancer cells.

The above evidence suggests that targeting the Wnt pathway may be one of the breakthroughs in alleviating drug resistance in gastric cancer patients.

## 6 Wnt signaling regulates EMT and metastasis in gastric cancer

The EMT is a reversible biological process that plays a crucial role in cancer cell metastasis. Ligands such as Wnt2, WNT5A, and WNT10B are involved in the EMT in gastric cancer (36, 75, 76). Chaperonin-containing T-complex protein 1ϵ subunit (CCT5) binds to E-cadherin and abrogates the interaction between E-cadherin and β-catenin, releasing β-catenin into the nucleus, leading to the EMT in gastric cancer cells and facilitating gastric cancer progression and lymphatic metastasis (77). Hsp90ab1, an isoform of heat shock protein 90, inhibits ubiquitin-mediated degradation of LRP5, leading to the upregulation of multiple targets of Wnt/β-catenin, activation of the Wnt pathway, and promotion of the EMT in gastric cancer cells (78). High mobility histone A2 (HMGA2) promotes the EMT, exacerbating GC metastasis through the Wnt/β-catenin pathway (79). Procalcitonin GA9 (PCDHGA9) may be a potential novel biomarker in GC and is closely related to the prognosis of GC patients. PCDHGA9 directly interacts with β-catenin and prevents the dissociation of β-catenin, thereby antagonizing the Wnt/β-catenin pathway and inhibiting EMT, which in turn inhibits invasive metastasis (9).The above evidence proves that it is feasible to delay the disease progression and improve the survival of gastric cancer patients by targeting the Wnt pathway.

## 7 Molecular agents targeting the Wnt pathway

In recent years, numerous inhibitors against the Wnt pathway have been developed, including small-molecule inhibitors (SMIs), monoclonal antibodies (mAbs), and peptide mimics, which can target Wnt ligands/receptors, β-catenin, and downstream target genes, respectively.

### 7.1 Targeting Wnt ligands/receptors

Hanaki et al. (80) showed that the anti-*WNT5A* polyclonal antibody pAb5a-5 prevented metastasis of gastric cancer cells by targeting *WNT5A* to inhibit the activation of Rac1 and the expression of laminin γ2. Dickkopf proteins (DKKs), which are endogenous Wnt ligand antagonists, showed hypermethylation and loss of inhibitory effects on Wnt proteins in gastric cancer. In 2021, Yang et al. (81) developed a chimeric 5/35 adenovirus-delivered Dickkopf-1 (Ad5/35-DKK1), which introduced DKK1 into CD44+ gastric cancer cells to promote the infection of GCSCs with the virus vector and the reduction in GCSC tumorigenicity. Porcupine (PORCN), for example, is a key molecule in inducing Wnt ligand secretion, and its inhibitors show great anticancer potential. The porcupine inhibitors (PORCNis) CGX1321 (NCT: 03507998), CGX1321 (with pembrolizumab) (NCT. 03507998) are all currently being evaluated in gastric cancer clinical trials to determine the maximum tolerated dose in patients with advanced gastrointestinal tumors (82). IWP (inhibitor of Wnt production) is a PORCNi that inhibits the growth of gastric cancer cells by downregulating the Wnt/β-catenin pathway (83). R-Spondin (RSPO) scavenges membrane Znrf3/Rnf43 and promotes activation of the Wnt pathway. The monoclonal antibody OMP131-R10 (rosmantuzumab) exhibits excellent safety and efficacy in patients with advanced solid tumor cancers (84). Hence, RSPO may be a potential therapeutic target. A recent study showed that PORCNi and anti-RSPO antibodies showed great potential as therapeutic targets for gastric cancer (85). Foxy-5, a peptide mimic of *WNT5A*, increases the expression level of *WNT5A* and prevents tumor metastasis in the breast and prostate cancer contexts (38).

MicroRNAs (miRNAs), as noncoding RNAs, can inhibit gene expression by binding to complementary mRNAs; thus, microRNAs are effective inhibitors. miRNAs targeting Wnt ligands are constantly being discovered. miR-132–3p and miR-140–5p can reduce the expression of *WNT1* and inhibit gastric cancer cell proliferation and invasion (86, 87); miR-491–5p can target and silence Wnt3a, inhibit the proliferation of gastric cancer cells and induce apoptosis (21). miR-876–5p and miR-26a-5p can reduce the expression of *WNT5A*, inhibit the proliferation and migration of gastric cancer cells and induce apoptosis (15).

FZD proteins, as receptors of Wnt ligands, are essential for the activation of the Wnt pathway. Therefore, researchers have inhibited the activated Wnt pathway in gastric cancer by FZD inhibitors. Fz7–21, an inhibitor of *FZD7*, inhibits the growth of gastric adenomas and is currently being evaluated in preclinical trials for patients with gastrointestinal tumors (88). In addition, knockdown of *FZD7* or treatment with vantictumab inhibited the growth of gastric adenomas. Vantictumab has been used in phase Ib clinical trials for advanced pancreatic, lung and breast cancers, a finding that demonstrates that it will be effective in treating patients with gastric cancer (89). The recombinant fusion protein ipafricept (OMP-54F28) mainly inhibits the binding of *FZD8* to the Wnt ligand and has been evaluated for its ability to inhibit tumor growth in Phase I clinical trials for patients with advanced solid tumors (90). The monoclonal antibody OMP-18R5 (Vantictumab) (NCT:01345201) targets the FZD receptor and reduces the clone-forming ability of gastric cancer cells (88). Salinomycin, an inhibitor of *LRP5/6*, inhibits the growth of GC by inhibiting Wnt signaling in CSCs, and salinomycin targeting Wnt/β-catenin pathway may have important clinical therapeutic value in gastric cancer (69). As an FDA-approved drug with relatively low toxicity, clonidine can simultaneously target multiple Wnt pathway targets, namely *FZD1*, *Dvl2*, and *LRP6*, and it has demonstrated greater inhibition of colorectal and breast cancers; studies with clonidine have also provided insights for research on the application of Wnt inhibitors in the treatment of gastric cancer (9).

### 7.2 Targeting beta-catenin and the beta-catenin/TCF transcription complex

M435–1279 is a novel ubiquitin-conjugating enzyme E2T (UBE2T) inhibitor that inhibits UBE2T-induced β-catenin accumulation in the nucleus, blocks the overactivation of the Wnt signaling pathway and effectively inhibits GC progression. More importantly, M435–1279 induces low cytotoxicity in normal gastric mucosal cells at a concentration that is effective against gastric cancer cells. Although the mechanism underlying M435–1279 action is clear, the results of clinical trials have not validated its effects (91). β-Catenin responsive transcription inhibitor 3/5 (iCRT3/5), which mainly blocks β-catenin-TCF4 interactions, is in the preclinical stage of gastric cancer treatment, and it can kill gastric cancer malignant cells and inhibit gastric cancer development (88). LF3 is a 4-thioureido-benzenesulfonamide derivative that profoundly inhibits the interaction between β-catenin and TCF4 and reduces the expression of GPX4, inducing iron-induced death in gastric cancer cells (29). In 2018, Wang et al. Found that XAV939, a small-molecule inhibitor (SMI), increases degradation of β-catenin by stabilizing Axin and inhibits gastric cancer invasion and metastasis (92).

### 7.3 Targeting β-catenin downstream target genes

In 2020, Chang et al. found that 2,4-Diaminoquinazoline (2,4-DAQ), a selective inhibitor of LEF1, inhibits the expression of AXIN2, MYC and LGR5 and suppresses the proliferation, migration and invasion of gastric cancer cells (93). The oral Compound E7386 selectively inhibited the interaction of β-catenin with the β-catenin transcriptional coactivator CREB-binding protein (CBP) and exerted high antitumor activity in a human gastric cancer xenograft tumor model (94).

In 2016, Feng et al. found that Pantoprazole, a proton pump inhibitor, inhibited the proliferation of gastric cancer cells and increased the therapeutic sensitivity of GC to 5-FU by inhibiting the EMT/β-catenin pathway (95). Ibuprofen, a commonly used antipyretic and analgesic, inhibited the Wnt/β-catenin pathway in gastric cancer stem cells to reduce their proliferation (96). Fiber-modified hexose chimeric recombinant lysosomal adenovirus targeting CAFs in TAMs specifically killed CAFs and inhibited the growth of gastric cancer cells (97). In summary, various molecular agents target the Wnt pathway, and monoclonal antibodies have received much attention from researchers because of their high specificity, which is in line with a precision therapy strategy (98).

Many molecular agents have been developed to target the Wnt pathway, but none of the FDA-approved drugs have been used in the clinical treatment of GC to date; the primary reason for this is the side-effects they produce, as they are, mainly toxic to bone. PORCN inhibitors cause loss of bone density and volume (99), and OMPs targeting FZD cause even more severe bone damage, which was the main reason that clinical trials to evaluate OMPs were discontinued. Monoclonal antibodies targeting FZD cause abdominal pain, diarrhea, and constipation (100), limiting their clinical applications. Second, the molecular structures of SMIs, mAbs and peptide mimics can mitigate the therapeutic effect. SMI show higher permeability and are suitable for oral administration, but they show low targeting specificity. mAbs have large molecular weights and a long half-life; however, they burden a patient’s liver and kidneys and are poorly tolerated. Although the emergence of peptide mimics solved the problems of permeability and tolerance of SMIs and mABs, due to their short half-life, larger doses are required to maintain therapeutic concentrations (38). In 2023, isoproterenol (2,6-diisopropylphenol), a commonly used intravenous anesthetic in clinical practice, significantly reduced the likelihood of cancer recurrence when used as an anesthetic for breast cancer surgery. Kaishuai Zhan et al. demonstrated that it can activate the Wnt/β-catenin pathway and inhibit the proliferation of gastric cancer cells (101).

Due to the complexity of the Wnt pathway, it is difficult to find targets that can be reached with high specificity and efficacy. The nonclassical Wnt pathway has been increasingly studied, but there are few drugs targeting the nonclassical Wnt pathway. Therefore, development of drugs targeting the nonclassical Wnt pathway is an emerging research direction.

## 8 Discussion

**Figure 2:**
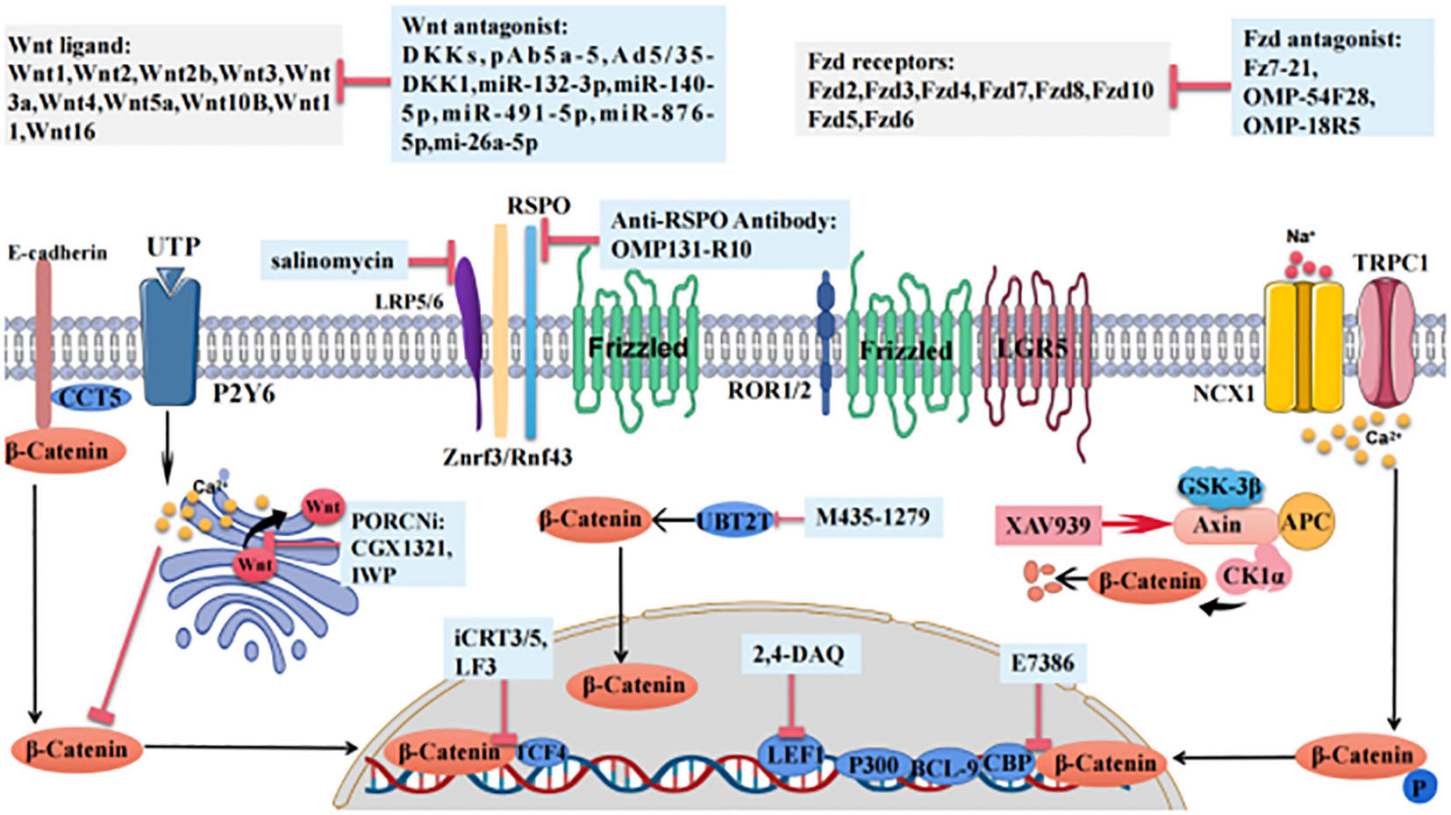
The roles of Wnt pathway members and Wnt-targeted agents in gastric cancer. Wnt ligands and FZD receptors are altered in gastric cancer. The background colors of the following various Wnt-targeted agents are shown in light blue boxes. DKKs and PORCNi are Wnt ligand antagonists, pAb5a-5 targets and inhibits WNT5A, Ad5/35-DKK1 delivers DKK1, miR-132–3p and miR-140–5p inhibit WNT1, miR-491–5p inhibits Wnt3a, and miR-876–5p and mi-26a-5p inhibit WNT5A. OMP-18R5 targets and inhibits the FZD receptor, Fz7–21 inhibits FZD7. OMP-54F28 inhibits FZD8. CCT5 inhibits β-catenin adhesion to E-cadherin, salinomycin inhibits LRP5/6, and OMP131-R10 inhibits the Znrf3-/Rnf43-scavenging effect of RSPO. M435–1279 inhibits UBT2T-induces β-catenin accumulation in the nucleus. XAV939 promotes β-catenin degradation. iCRT3/5 and LF3 inhibit the interaction of β-catenin and TCF4. 2,4-DAQ inhibits LEF1, and E7386 inhibits the interaction between β-catenin and CBP.

Abnormal activation of Wnt signaling plays important roles in the occurrence, development and chemotherapy resistance of gastric cancer. Targeting the Wnt pathway is a great strategy for the treatment of GC (**Figure 2**). An increasing number of small molecules and biological agents targeting the Wnt pathway have been entered into clinical trials for patients with GC. In view of the unique role of the non-classical Wnt pathway in gastric cancer, more specific inhibitors can be developed. Whether Wnt pathway inhibitors can be used in the clinical treatment of GC in the future depends on how the problems with targeting, efficacy and patient tolerance by Wnt pathway inhibitors are solved.

## Author contributions

RH: Writing – original draft. JY: Writing – review & editing. YZ: Supervision, Writing – review & editing. RG: Supervision, Writing – review & editing.
